# Sleep problems and insomnia are common and associated with pain intensity, number of comorbidities and analgesic use in patients with knee and hip osteoarthritis: a cross-sectional study using data from the good life with osteoarthritis in Denmark (GLA:D^®^) registry

**DOI:** 10.1007/s00296-025-05878-4

**Published:** 2025-04-25

**Authors:** Jonas B. Thorlund, Eivind S. Skarpsno, Jonas J. Vestergaard, Søren T. Skou, Dorte T. Grønne, Ewa M. Roos, Henrik B. Vaegter

**Affiliations:** 1 Center for Muscle and Joint Health, Department of Sports Science and Clinical Biomechanics, University of Southern Denmark, Campusvej 55, Odense, 5230 Denmark; 2 Research Unit for General Practice, Department of Public Health, University of Southern Denmark, Odense, Denmark; 3 Department of Public Health and Nursing, Norwegian University of Science and Technology, Trondheim, Norway; 4 Department of Neurology and Clinical Neurophysiology, St. Olavs Hospital, Trondheim, Norway; 5 The Research and Implementation Unit PROgrez, Department of Physiotherapy and Occupational Therapy, Naestved-Slagelse- Ringsted Hospitals, Slagelse, Denmark; 6 Pain Research Group, Pain Center, Odense University Hospital, Odense, Denmark; 7 Department of Clinical Research, Faculty of Health Sciences, University of Southern Denmark, Odense, Denmark

**Keywords:** Sleep disturbance, Musculoskeletal health, Chronic pain, Insomnia, Surveys and questionnaires

## Abstract

To assess the difference in prevalence of sleep problems and insomnia in patients with knee or hip osteoarthritis (OA), and explore characteristics associated with sleep problems and insomnia. We included 8,162 knee/hip OA patients enrolled in supervised exercise and patient education through the Good Life with osteoArthritis in Denmark (GLA:D^®^) program. We assessed presence of sleep problems (yes/no), followed by the Insomnia Severity Index 3-item (ISI-3) questionnaire among those with sleep problems (Insomnia: ISI-3 score ≥ 7). Characteristics associated with sleep problems/insomnia was estimated for knee and hip OA patients separately (prevalence ratios [PR]). In total, 68% (*n* = 3,539) and 64% (*n* = 1,807) of knee and hip OA patients reported sleep problems, respectively, corresponding to a PR of 1.06 (95% CI 1.03 to 1.10). Prevalence of insomnia was 17% (*n* = 943) and 20% (*n* = 528) for those with knee and hip OA, respectively (PR 1.18 [95% CI 1.07 to 1.30]). Large overlap between characteristics associated with sleep problems and insomnia were observed. Characteristics most strongly associated with higher prevalence of insomnia were pain intensity ≥40 mm VAS (knee: PR 2.39 [95% CI 2.08 to 2.74]; hip: PR 2.54 [95% CI 2.10 to 3.07], a high number of comorbidities, and analgesic use in both patients with knee and hip OA. Sleep problems and insomnia are highly prevalent among primary care patients with knee and hip OA, and slightly more common in hip OA patients. Prevalence of insomnia was substantially higher among patients with more comorbidities, higher pain intensity and analgesic use.

## Introduction

More than 50% of people with chronic pain suffer from symptoms of comorbid insomnia [1, 2]. Insomnia is a sleep disorder characterized by difficulties in initiating or maintaining sleep, and therefore frequently associated with daytime complaints such as sleepiness, fatigue, somatic symptoms (e.g., head or body aches), mood disturbances, and compromised daily functioning​ [3].

Previous research has reported on the prevalence of sleep problems and insomnia in the sub population of chronic pain patients with osteoarthritis (OA) [4–8]. In these studies, sleep problems were more common in patients with OA compared to the general population [4, 5, 8], and in the Johnston County Osteoarthritis Project approximately 56% of OA patients had at least one current symptom of insomnia [5]. Furthermore, characteristics such as older age, depression, higher BMI and higher pain levels were associated with insomnia among patients with OA [4, 6, 9, 10].

A limitation of most previous research is that they have not provided data on sleep problems and insomnia for patients with knee or hip OA separately [4–10]. This limit the ability to ascertain differences in prevalence of sleep problems and insomnia in patients with knee or hip OA and if patient characteristics associated with sleep problems and insomnia are joint specific. Such differences may exist, as patients with knee and hip OA have been reported to differ in several ways. For example, patients with hip OA have been reported to describe more intense pain than patients with knee OA and also to use more analgesics [11].

Therefore, the aim of this study was to assess the difference in prevalence of sleep problems and insomnia in patients with knee or hip OA using data from a large Danish primary care cohort, and to explore the specific characteristics of patients with these comorbid sleep issues in patients with knee or hip OA, respectively.

## Materials and methods

For this study we used baseline data (collected before starting the education and exercise program) from the Good Life with osteoArthritis in Denmark (GLA:D^®^) registry. GLA:D^®^ is a Danish nationwide initiative implementing supervised exercise therapy and patient education according to international clinical treatment guidelines for patients with knee and hip OA [12]. The GLA:D^®^ treatment program consists of 2–3 sessions of patient education and 12 supervised sessions of group-based neuromuscular exercise (60 min. per session, twice weekly), which is delivered by certified health professionals.

### Patients

Patients with a clinical diagnosis of knee or hip OA are eligible to participate in the GLA:D^®^ program. This is evaluated by the treating physiotherapists who is trained in giving a clinical diagnosis of knee or hip OA. Radiographs are not needed to diagnose OA according to international guidelines [13], but about 80% of patients in the GLA:D^®^ cohort have had an X-ray, with > 90% of these self-reporting to have radiographic knee and/or hip OA. Participants are excluded from participation in GLA:D^®^ if having another reason than OA for their knee/hip joint problems such as tumor or an inflammatory joint disease, other symptoms that are more pronounced than the knee/hip problems (e.g. generalized pain, or fibromyalgia), or do not understand Danish [12].

The Ethics Committee of the North Denmark Region waived the need for ethical approval for the GLA:D^®^ registry [12]. The GLA:D^®^ registry has been approved by the Danish Data Protection Agency (SDU; 10.084). Before study participation, participants were informed about the data registration in GLA:D^®^. According to the Danish Data Protection Act, patient consent was not required as personal data were processed exclusively for research and statistical purposes.

### Self-reported sleep problems and insomnia

Questions on sleep problems were introduced in the GLA:D^®^ registry on January 6th 2023 and we used data from patients enrolled up until September 4th 2024. As the questions on the Insomnia Severity Index (ISI) questionnaire [14] assume that a respondent has some degree of current sleep problems, we used a two-step approach to assess sleep problems and insomnia. First, patients were asked ‘Have you experienced sleep problems within the last two weeks? (E.g. trouble falling asleep, slept bad/restless, awakened multiple times, and had difficulties falling asleep again, awakened too early without being able to fall asleep again or not felt rested when you woke up)´, with response options ‘yes’ or ‘no’. Patients responding ‘yes’ to the first question were defined as having ‘sleep problems’. Patients who reported sleep problems received three additional questions from the Insomnia Severity Index short version (ISI-3) [15]: For each of the 3 questions below, please indicate the answer corresponding most accurately to your sleep patterns in the last 2 weeks 1) ‘How satisfied/dissatisfied are you with your current sleep pattern?’; 2) ‘To what extent do you consider your sleep problem to interfere with your daily functioning (E.g., daytime fatigue, ability to function at work/daily chores, concentration, memory, mood)?’; and 3) ‘How worried/distressed are you about your current sleep problem?’. Response options for each question in the ISI-3 is on a five-point Likert-scale, ranging from zero, indicating the least dissatisfaction, interference, and worriedness, to four, indicating the highest. The response scores were added together, for a total ISI-3 score (range: 0–12 points). Patients with an ISI-3 total score of 7 or above, were categorized as having insomnia. The ISI-3 questionnaire has been shown to have high sensitivity (0.94–0.97) and specificity (0.88–0.91) for identifying clinically significant insomnia [15].

### Patient characteristics

At enrolment, patients self-reported the following into the GLA:D^®^ registry: age; sex (male/female); most symptomatic joint (knee or hip); height and weight (to calculate body mass index - BMI [kg/m^2^]).

#### Educational level

Patients were asked to self-report their highest level of education. Highest self-reported educational level was then classified as either ‘low’ (No education completed/Primary school/Secondary school) or ‘high’ (vocational education/Short term education/Middle term education/Long term education).

#### No. of comorbidities (0–12)

Patients were asked to self-report, if they had one or more comorbidities from a list of 12 comorbidities: Hypertension, cardiovascular diseases, lung diseases, diabetes, stomach diseases, kidney/liver diseases, blood diseases, cancer, rheumatoid arthritis, neurological disorders, depression, or other medical diseases.

#### Level of physical activity

Was assessed using the Danish version of the University of California, Los Angeles (UCLA)-activity scale [16]. The UCLA-activity scale is a 10-point scale, ranging from one indicating patients to be completely inactive and, dependent on others without the capability to leave their residence, to ten, indicating that patients regularly participate in impact sports such as jogging, soccer or handball, or performing heavy labour, or backpacking. For analyses patients were categorized as having either low (UCLA score ≤5) or high (UCLA score ≥6 physical activity level using previously defined cut-off values [17].

#### Analgesic use

At the initial visit, patients were asked by a clinician whether they had used pain medication, during the last two weeks (yes/no).

#### Pain intensity

Average pain intensity in the most symptomatic joint (knee or hip) during the last week was reported using a visual analogue scale (VAS 0–100 mm), with 0 representing no pain and 100 extreme pain. For analyses we categorized patients according to pain intensity < 40 mm or ≥40 mm). This cut-off was chosen, as it previously has been used in clinical trials to identify patients with significant knee or hip pain [18, 19].

### Statistics

Descriptive statistics stratified by knee or hip OA were reported as means with SD, median with interquartile range or numbers with percentages as appropriate. Firstly, we compared the prevalence of sleep problems and insomnia between patients with knee and hip OA, respectively, as the prevalence ratio, which was estimated by Poisson regression with robust standard errors adjusted for age (continuous) and sex (women, men). Secondly, we conducted separate analyses for patients with knee or hip OA, respectively, to assess characteristics associated with sleep problems and insomnia. In specific, prevalence ratios were estimated using Poisson regression with robust standard errors for each of the following variables: age (< 55, 55–64, 65–74, ≥ 75); sex (women, men); BMI (< 25, 25–29, ≥ 30); education level (low, high); physical activity level (low, high); comorbidities (none, 1–2, 3–4, ≥ 5); analgesic use (yes, no); and pain intensity (< 40 mm, ≥ 40 mm). The first category in each variable served as the reference group. STATA version 18.0 was used for all statistical analyses, and we considered *p* < 0.05 as statistically significant.

## Results

A total of 8,1622 patients were included in this study, with 5,517 patients having knee OA (68%). Patients were on average 67 years old, most were women (67%) and the average BMI was 28.6 kg/m^2^. The average pain intensity during the last week was moderate (~ 45 mm on a 0-100 VAS scale) and 3 out of 4 of patients reported having comorbidities (Table 1).

**Table 1:** Patient characteristics

	Knee OA (*n* = 5,517)	Hip OA (*n* = 2,645)	Total (*n* = 8,162)
Age, mean (SD)	66.4 (9.6)	67.3 (9.6)	66.7 (9.6)
Women, no. (%)	3,600 (65%)	1,826 (69%)	5,426 (67%)
BMI, mean (SD)*	29.1 (5.7)	27.5 (4.9)	28.6 (5.5)
High education level, n (%)^a^:	4,719 (86%)	2,244 (85%)	6,963 (85%)
High physical activity level, n (%)^b^:	2,771 (50%)	1,427 (54%)	4,198 (51%)
Comorbidities,* n* (%)^*c*^:			
None	1,487 (27%)	685 (26%)	2,172 (27%)
1–2	3,121 (57%)	1,484 (56%)	4,605 (56%)
3–4	801 (15%)	416 (16%)	1,217 (15%)
5 or more	108 (2%)	60 (2%)	168 (2%)
Analgesic use, n (%)	3,370 (61%)	1,808 (68%)	5,178 (63%)
VAS pain last week, mean (SD)	44.6 (23.1)	45.6 (22.4)	44.9 (22.9)

Percentages may not add up to 100% due to rounding

* Missing data: BMI, *n* = 5; Radiographic OA, *n* = 1,003

^a^ Highest self-reported educational level was classified as either low (No education completed/Primary school/Secondary school) or high (vocational education/Short term education/Middle term education/Long term education)

^b^ UCLA score of 5 or lower = low; UCLA score of 6 or higher = high. The UCLA-activity scale ranges from 0–10 (0 = wholly inactive and 10 = regularly participation in impact sports)

^c^ No. of comorbidities among: hypertension, cardiovascular diseases, lung diseases, diabetes, stomach diseases, kidney/liver diseases, blood diseases, cancer, rheumatoid arthritis, neurological disorders, depression, or other medical diseases

Sleep problems were reported among 68% (95% CI 67 to 70) of the patients with hip OA and 64% (95% CI 63 to 65) of the patients with knee OA, corresponding to a prevalence ratio of 1.06 (95% CI 1.03 to 1.10). Insomnia was reported among 20% (95% CI 18 to 22) of patients with hip OA and 17% (95% CI 16 to 18) of patients with knee OA, yielding a prevalence ratio of 1.18 (95% CI 1.07 to 1.30) (Table 2).

**Table 2:** Prevalence of sleep problems and insomnia in patients with knee or hip OA

		Sleep problems^*a*^		Insomnia^*b*^	
	*n*	No. of prevalent cases (%)	Prevalence ratio^c^ (95% CI)	No. of prevalent cases (%)	Prevalence ratio^c^ (95% CI)
Knee OA	5,517	3,539 (64%)	Ref.	943 (17%)	Ref.
Hip OA	2,645	1,807 (68%)	1.06 (1.03 to 1.10)	528 (20%)	1.18 (1.07 to 1.30)

^a^ Patients with sleep problems (i.e. replying ‘yes’ to the entry question about sleep problems)

^b^ Patients with insomnia (i.e. patients with an ISI-3 score ≥7 among patients with sleep problems)

^c^ Adjusted for age (continuous) and sex (women, men)

Several patient characteristics were associated with prevalence of sleep problems and insomnia. Prevalence ratios generally indicated that older age was associated with lower prevalence of sleep problems and insomnia in patients with knee or hip OA. Similarly, the prevalence of both sleep problems and insomnia was higher in women in patients with knee or hip OA. BMI was only associated with higher prevalence of insomnia in patients with high BMI (≥30), which was observed in both knee and hip OA patients (Tables 3 and 4).

**Table 3:** Characteristics associated with prevalence and prevalence ratio of sleep problems and insomnia among patients with ***knee OA*** (*n* = 5,517)

	*n*	Sleep problems^a^		Insomnia^b^	
		Prevalence (95% CI)	Prevalence ratio (95% CI)^c^	Prevalence (95% CI)	Prevalence ratio (95% CI)^d^
**Age:**					
<55 yrs	627	72% (68 to 75)	Ref.	22% (19 to 26)	Ref.
55–64 yrs	1,621	70% (67 to 72)	0.98 (0.92 to 1.04)	21% (19 to 23)	0.95 (0.80 to 1.13)
65–74 yrs	2,060	63% (61 to 65)	**0.90 (0.85 to 0.95)**	15% (14 to 17)	**0.69 (0.58 to 0.83)**
≥75 yrs	1,209	54% (51 to 57)	**0.77 (0.72 to 0.83)**	12% (11 to 15)	**0.57 (0.46 to 0.70)**
**Sex**:					
Male	1,917	53% (51 to 55)	Ref.	14% (13 to 16)	Ref.
Female	3,600	70% (69 to 72)	**1.30 (1.24 to 1.37)**	19% (17 to 20)	**1.27 (1.12 to 1.45)**
**BMI**:					
<25	1,311	63% (60 to 66)	Ref.	15% (13 to 17)	Ref.
25–29	2,164	63% (61 to 65)	1.01 (0.96 to 1.07)	15% (14 to 17)	1.04 (0.88 to 1.23)
≥30	2,039	67% (65 to 69)	1.03 (0.97 to 1.08)	20% (19 to 22)	**1.27 (1.08 to 1.49)**
**Education level**:					
Low	798	63% (60 to 67)	Ref.	17% (15 to 20)	Ref.
High	4,719	64% (63 to 66)	1.00 (0.95 to 1.06)	17% (16 to 18)	0.95 (0.81 to 1.12)
**Physical activity level**:					
Low	2,746	67% (65 to 68)	Ref.	21% (19 to 22)	Ref.
High	2,771	62% (60 to 63)	**0.94 (0.91 to 0.98)**	13% (12 to 15)	**0.66 (0.59 to 0.75)**
**Comorbidities**:					
0	1,487	63% (60 to 65)	Ref.	14% (12 to 16)	Ref.
1–2	3,121	63% (62 to 65)	1.04 (1.00 to 1.09)	16% (15 to 18)	**1.30 (1.11 to 1.50)**
3–4	801	68% (65 to 72)	**1.15 (1.08 to 1.23)**	23% (20 to 26)	**1.95 (1.63 to 2.34)**
≥5	108	75% (66 to 83)	**1.28 (1.15 to 1.44)**	35% (26 to 45)	**2.97 (2.24 to 3.93)**
**Analgesic use**:					
No	2,149	53% (51 to 56)	Ref.	11% (10 to 12)	Ref.
Yes	3,370	71% (69 to 72)	**1.30 (1.25 to 1.36)**	21% (20 to 22)	**1.90 (1.65 to 2.18)**
**Pain intensity (VAS)**:					
<40 mm	2,432	56% (54 to 58)	Ref.	9% (8 to 11)	Ref.
≥40 mm	3,085	71% (69 to 72)	**1.24 (1.19 to 1.29)**	23% (22 to 25)	**2.39 (2.08 to 2.74)**

Prevalence ratios with 95% CI

^a^ Patients with sleep problems (i.e. replying ‘yes’ to the entry question about sleep problems)

^b^ Patients with insomnia (i.e. patients with an ISI-3 score ≥7 among patients with sleep problems)

^c^ Adjusted for age (continuous) and sex (women/men), reference = patients without sleep problems

^d^ Adjusted for age (continuous) and sex (women/men), reference = patients without insomnia

**Table 4:** Characteristics associated with prevalence and prevalence ratio of sleep problems and insomnia among patients with ***hip OA*** (*n* = 2,645)

	*n*	Sleep problems^a^		Insomnia^b^	
		Prevalence (95% CI)	Prevalence ratio (95% CI)^c^	Prevalence (95% CI)	Prevalence ratio (95% CI)^d^
**Age:**					
<55 yrs	272	79% (74 to 84)	Ref.	28% (23 to 34)	Ref.
55–64 yrs	680	77% (73 to 80)	0.98 (0.91 to 1.05)	25% (22 to 29)	0.91 (0.72 to 1.15)
65–74 yrs	1,024	66% (63 to 69)	**0.85 (0.79 to 0.91)**	17% (15 to 20)	**0.63 (0.50 to 0.80)**
≥75 yrs	669	59% (55 to 63)	**0.76 (0.70 to 0.83)**	15% (13 to 18)	**0.56 (0.43 to 0.73)**
**Sex**:					
Male	819	55% (52 to 59)	Ref.	16% (13 to 18)	Ref.
Female	1,826	74% (72 to 76)	**1.32 (1.24 to 1.42)**	22% (20 to 24)	**1.36 (1.14 to 1.63)**
**BMI**:					
<25	871	70% (67 to 73)	Ref.	18% (16 to 21)	Ref.
25–29	1,065	66% (63 to 69)	0.97 (0.91 to 1.03)	19% (16 to 21)	1.04 (0.86 to 1.26)
≥30	707	70% (66 to 73)	0.99 (0.93 to 1.05)	24% (21 to 27)	**1.27 (1.05 to 1.54)**
**Education level**:					
Low	401	64% (59 to 69)	Ref.	21% (17 to 25)	Ref.
High	2,244	69% (67 to 71)	1.06 (0.99 to 1.15)	20% (18 to 22)	0.94 (0.77 to 1.16)
**Physical activity level**:					
Low	1,218	69% (67 to 72)	Ref.	24% (21 to 26)	Ref.
High	1,417	68% (65 to 70)	0.97 (0.92 to 1.02)	17% (15 to 19)	**0.70 (0.60 to 0.82)**
**Comorbidities**:					
0	685	65% (61 to 69)	Ref.	14% (12 to 17)	Ref.
1–2	1,484	69% (67 to 72)	1.11 (1.04 to 1.18)	20% (18 to 22)	**1.59 (1.28 to 1.96)**
3–4	416	70% (66 to 75)	**1.18 (1.08 to 1.28)**	29% (24 to 33)	**2.50 (1.96 to 3.18)**
≥5	60	70% (57 to 81)	1.19 (1.00 to 1.40)	28% (17 to 41)	**2.53 (1.64 to 3.91)**
**Analgesic use**:					
No	837	59% (56 to 62)	Ref.	13% (11 to 15)	Ref.
Yes	1,808	73% (70 to 75)	**1.20 (1.13 to 1.28)**	23% (21 to 25)	**1.76 (1.45 to 2.14)**
**Pain intensity (VAS)**:					
<40 mm	1,127	60% (57 to 63)	Ref.	10% (9 to 12)	Ref.
≥ 40 mm	1,518	74 (72 to 77)	**1.76 (1.45 to 1.31)**	27% (25 to 29)	**2.54 (2.10 to 3.07)**

Prevalence ratios with 95% CI

^a^ Patients with sleep problems (i.e. replying ‘yes’ to the entry question about sleep problems)

^b^ Patients with insomnia (i.e. patients with an ISI-3 score ≥7 among patients with sleep problems)

^c^ Adjusted for age (continuous) and sex (women/men), reference = patients without sleep problems

^d^ Adjusted for age (continuous) and sex (women/men), reference = patients without insomnia

Generally, prevalence ratios indicated that higher physical level was associated with lower prevalence of sleep problems, though not statistically significant for hip patients. This association was even stronger for insomnia, showing a prevalence ration of 0.66 (95% CI 0.59 to 0.75) for knee OA and 0.70 (95% CI 0.60 to 0.82) for hip OA. Education level was not observed to be associated with sleep problems or insomnia (Tables 3 and 4).

In both patients with knee and hip OA the prevalence of sleep problems was higher among patients with more comorbidities. For insomnia this association was even stronger with prevalence ratios of 2.97 (95% CI 2.24 to 3.93) for knee OA patients and 2.53 (95% CI 1.64 to 3.91) for hip OA patients with 5 or more comorbidities. Analgesic use was also associated with sleep problems in knee and hip OA patients, and this association was even stronger for insomnia with prevalence ratios of 1.90 (95% CI 1.65 to 2.74) and 1.76 (95% CI 1.45 to 2.14) for knee and hip OA patients, respectively. Finally, VAS pain intensity ≥40 mm was associated with sleep problems, and this association was even stronger for insomnia with a prevalence ratio of 2.39 (95% CI 2.08 to 2.74) for knee and 2.54 (95% CI 2.10 to 3.07) for hip OA (Tables 3 and 4).

## Discussion

In a large cohort of patients with knee and hip OA treated in primary care, 64% and 68% of patients with knee or hip OA reported sleep problems, and 17% and 20% of patients with knee or hip OA had insomnia, respectively. Large overlap was observed between characteristics associated with higher prevalence of sleep problems and insomnia in both patients with knee and hip OA. Patient characteristics most strongly associated with higher prevalence of insomnia were VAS pain intensity ≥40 mm, analgesic use and high number of comorbidities in both patients with knee and hip OA.

Insomnia is common in people with chronic pain [1], and our study confirm previous studies reporting that this also apply to patients with knee and hip OA. In the Johnston County Osteoarthritis Project [5], 56% of OA patients experienced at least one symptom of insomnia,​ whereas 65% responded positive to the entry question about sleep problems in our study. Direct comparison of prevalence estimates between studies is difficult as methods used for assessment of sleep problems varies, and presence of insomnia is defined differently across studies. In our study, 17% and 20% of knee and hip OA patients, respectively, had insomnia defined as an ISI-3 score of 7 or more [15]. In comparison, 53% of a sample of US veterans (*n* = 300) with knee or hip OA had insomnia defined as an ISI score of 10 or more using the full 7-question ISI scale [6, 20]. The 10-point cutoff on the full ISI scale (range 0–24), likely represent less symptomatic insomnia than in our study using a 7 points cutoff on the ISI-3 scale (range 0–12), and veterans may also represent a population with higher prevalence of insomnia.

Despite the difficulty in comparing prevalence estimates, it is clear that insomnia is common among patients with knee and hip OA. Our study extends on previous research in Rheumatology International and other journals by reporting estimates for knee and hip OA separately from a large cohort of patients [4–10]. Although our data suggest that sleep problems and insomnia are more prevalent among patients with hip than knee OA, we can only speculate on reasons for this. Previous research has reported that pain may be more intense in patients with hip than knee OA, which in turn may impact sleep [11]. It should be noted that the prevalence differences between patients with knee and hip OA for sleep problems and insomnia were only 4% and 3%, respectively.

We observed large overlap between patient characteristics associated with sleep problems and insomnia in patients with knee or hip OA. Our results suggested that older age was associated with lower prevalence of sleep problems and insomnia in both knee and hip OA patients. This finding is similar to some studies [5, 6], but not others [8], which may be partly explained by differences in the sub populations studied. We also observed that female sex, more comorbidities, analgesic use and VAS pain intensity ≥40 mm was associated with higher prevalence of sleep problems and insomnia in both knee and hip OA patients, which is consistent with previous studies reporting estimates from mixed patients with both knee and hip OA [8, 10, 21]. Previous studies on mixed populations of knee and hip OA patients have reported estimates pointing in the direction of an association between sleep problems and high BMI/obesity, but with 95% CI overlapping 1.00 [6, 10]. We observed that obesity was associated with a 27% higher prevalence of insomnia in both knee and hip OA patients, which may be partly due to the large sample size in the present study, allowing us to detect this difference. Lastly, we observed that higher physical activity level was associated with both lower prevalence of sleep problems and insomnia in patients with knee OA, but this was only observed for insomnia in patients with hip OA. We are not aware of previous research reporting on the association between physical activity and sleep in patients with knee or hip OA. However, previous research has suggested that exercise can improve sleep quality in older adults, which may explain this finding [22]. On the other hand, it has been suggested that the relationship between exercise/physical activity and sleep may be bi-directional as poor sleep also has been reported to contribute to low physical activity levels [23].

The cross-sectional nature of this study limits the possibility of drawing causal inferences from the results. Furthermore, due to the potential bi-directional relationship between sleep and physical activity [23] and the ample evidence of a bi-directional relationship between sleep problems/insomnia and pain [24] we only adjusted for age and sex in our models, and refrained from using large confounder adjusted models to estimate prevalence ratios as such models were likely to cause spurious associations.

The present study, and previous research highlight that sleep problems and insomnia are common issues in patients with knee and hip osteoarthritis. Screening for insomnia in patients with knee and particular hip OA should be considered in clinical practice, as a potential target for management to improve overall care. Cognitive Behavioral Therapy for Insomnia (CBTi) is first-line treatment for insomnia [25], and CBTi has also been shown to improve sleep and fatigue, and to a lesser extent pain in patients with OA and comorbid insomnia [26]. However, if CBTi can be combined with guideline recommended OA care is not known and has been flagged as a target for future research [27].

Our study has some limitations. First, we relied on the short form ISI-3 questionnaire to assess insomnia and not the original 7-item ISI questionnaire or a clinical diagnosis, however the ISI-3 screening tool has shown high sensitivity and specificity to identify patients with clinical significant insomnia (i.e. ISI-7 score of 15 or more) [15]. As many types of information is collected from patients in the GLA:D^®^ registry, we used the ISI-3 to keep the response burden to patients at a minimum. Second, our prevalence estimates of sleep problems and insomnia in OA may be underestimated as our population was fairly active (i.e. median physical activity level corresponded to ‘regularly participation in moderate activities’) [16]. Third, while we included a crude measure of analgesic use (yes/no) we did not have information about the amount of analgesics used or use of sleep medicine available from prescription registries, and are therefore unable to assess the influence of this on our estimates. Finally, despite the large sample size, our findings may not apply to all patients with knee and hip OA, as patients in the present study represent patients actively seeking primary care. In Denmark, visits to the GP are free while physiotherapy is associated with a patient fee for most.

In conclusion, both sleep problems (68% vs. 64%) and insomnia (20% vs. 17%) were more common in patients with hip OA than knee OA. There was large overlap between patient characteristics associated with higher prevalence of sleep problems and insomnia in patients with knee and hip OA. The characteristics most strongly associated with higher prevalence of insomnia were a high number of comorbidities, VAS pain intensity ≥40 mm and analgesic use in both patients with knee and hip OA.

## Data Availability

Access to the data is restricted and may be obtained solely through the corresponding author upon reasonable request. The data is not publicly available due to the data protection legislation as the data contains information that could compromise the privacy of the research participants.

## Declarations

### Related work and use of AI

Part of the data was presented as an abstract at the World Congress on Osteoarthritis (OARSI) 2024: Osteoarthritis Cartilage 2024;32(Suppl 1):S194. AI was used for checking of spelling, grammar and language editing.

### Disclaimer

No part of this paper has been copied or published elsewhere in whole or in part.
